# Immune-related cardiovascular toxicities of PD-1/PD-L1 inhibitors in solid tumors: an updated systematic review and meta-analysis

**DOI:** 10.3389/fimmu.2024.1255825

**Published:** 2024-01-22

**Authors:** Chi Zhang, Fengtao Wei, Wenhan Ma, Jingbo Zhang

**Affiliations:** Department of Cardiology, The Second Hospital of Shandong University, Jinan, Shandong, China

**Keywords:** PD-1/PD-L1 inhibitors, solid tumors, cardiotoxicity, vascular toxicity, meta-analysis

## Abstract

**Purpose:**

The objective of this study was to investigate the risk of cardiovascular toxicities related to PD-1/PD-L1 inhibitors in solid tumors.

**Methods:**

A literature search was performed following the participants, interventions, comparisons, outcomes, and study design (PICOS) principles, and the study adhered to the Preferred Reporting Items for Systematic Reviews and Meta-Analyses (PRISMA) guidelines. Data analysis was conducted using Review Manager version 5.4.

**Results:**

This meta-analysis included 69 randomized controlled trials (RCTs) divided into five groups based on the treatment regimens: PD-1/PD-L1 + chemotherapy versus chemotherapy, PD-1/PD-L1 versus chemotherapy, PD-1/PD-L1 versus placebo, PD-1/PD-L1 + CTLA-4 versus PD-1/PD-L1 and PD-1/PD-L1 + CTLA-4 versus chemotherapy. Compared to chemotherapy treatment alone, PD-1/PD-L1 +chemotherapy significantly increased the risk of hypertension [all-grade (OR = 1.27, 95% CI [1.05, 1.53], p = 0.01); grade 3–5 (OR = 1.36, 95% CI [1.04, 1.79], p = 0.03)], hypotension [all-grade (OR = 2.03, 95% CI [1.19, 3.45], p = 0.009); grade 3–5 (OR = 3.60, 95% CI [1.22, 10.60], p = 0.02)], arrhythmia [all-grade (OR = 1.53, 95% CI [1.02, 2.30], p = 0.04); grade 3–5 (OR = 2.91, 95% CI [1.33, 6.39], p = 0.008)] and myocarditis [all-grade (OR = 2.42, 95% CI [1.06, 5.54], p = 0.04)]. The risk of all-grade hypotension (OR = 2.87, 95% CI [1.26, 6.55], p = 0.01) and all-grade arrhythmia (OR = 2.03, 95% CI [1.13, 3.64], p = 0.02) significantly increased when treated with PD-1/PD-L1 inhibitors compared to the placebo. The risks of cardiovascular toxicities are significantly higher with PD-1+CTLA-4 compared to PD-1 alone (OR = 2.02, 95% CI [1.12, 3.66], p = 0.02).

**Conclusion:**

PD-1/PD-L1 inhibitor leads to an increased risk of cardiovascular toxicities, especially hypertension, hypotension, arrhythmia, and myocarditis.

## Introduction

In recent years, the programmed cell death 1/programmed cell death 1 ligand 1 (PD-1/PD-L1) inhibitor has been used as an immunotherapy and has led to substantial advancements in the prognosis of diverse cancer types (1). It can enhance the immune response by blocking the inhibitory signal of the T cell response and exerting anti-tumor effects (2). However, the enhanced destructive effect of T cells can also damage normal cells and tissues. Clinicians are becoming aware of its adverse effects on almost all organ types (3). Adverse effects often include immune-related pneumonitis, liver damage, endocrine organ abnormalities, and adverse skin reactions (4). Although cardiovascular toxicities, such as myocarditis, arrhythmia, blood pressure abnormalities, and heart failure, are uncommon, their prognoses are poor (5, 6). Therefore, additional attention should be paid to cardiovascular toxicity.

PD-1/PD-L1 inhibitors are currently recommended in various therapeutic combinations. Previous reviews and meta-analyses have summarized cardiovascular toxicities associated with different treatment regimens (7, 8). The completion of more clinical trials may have affected the original analysis results. The original topic that could not be analyzed because of insufficient data may have to be reoperated and completed. Therefore, given that cardiovascular toxicities are now considered major determinants of prognosis (9), it is necessary to conduct a new meta-analysis for this study. This will further guide the antitumor treatment of patients with solid tumors.

## Materials and methods

### Search strategy and selection criteria

This study was consistent with the Preferred Reporting Items for Systematic Reviews and Meta-Analyses (PRISMA) guidelines (10). Randomized controlled trials (RCTs) on solid tumors with cardiovascular toxicities published between July 2013 and May 2023 were searched based on the principle of PICOS (participants, interventions, comparisons, outcomes, and study design). The following medical subject heading (MeSH) terms were used: nivolumab, pembrolizumab, atezolizumab, tislelizumab, penpulimab, avelumab, durvalumab, camrelizumab, Opdivo, Bavencio, Keytruda, Imfinzi, AK105, MPDL3280A, Tecentriq, MK-3475, and BMS 963558. RCTs mentioned in the relevant reviews and references were also searched to avoid missing data. Five individuals were selected for literature search and data extraction. All conflicts were jointly discussed and resolved by the corresponding author.

The following selection criteria were used: 1) RCTs published between July 2013 and May 2023; 2) participants diagnosed with solid tumors treated with at least one PD-1 or PD-L1 inhibitor; 3) clinical trials reporting all-grade or grade 3–5 adverse effects; 4) research published in English. The exclusion criteria were as follows: 1) no treatment with PD-1/PD-L1; 2) non-RCT studies; 3) RCTs not involving cardiovascular toxicities; 4) single-arm studies without a control group.

### Data extraction

Five individuals independently obtained the following baseline information from the included studies: year of publication, name of the first author, name of the study, national clinical trial (NCT) number, treatment lines, names of tumors, names of drugs, treatment arms, and the total number of people included in each study.

### Publication bias and quality assessments

The Cochrane Collaboration tool was used to evaluate the risk of bias in the RCTs and funnel plots were used to evaluate publication bias (11). Seven sources of bias were evaluated in each RCT: random sequence generation (selection bias), allocation concealment (selection bias), blinding of participants and personnel (performance bias), blinding of outcome assessment (detection bias), incomplete outcome data (attrition bias), and selective reporting (reporting bias). Each domain was independently assigned a ‘high’, ‘low’, or ‘unclear’ risk of bias by all authors, with disagreements adjudicated by the corresponding author.

### Heterogeneity assessment and statistical analysis

Review Manager (RevMan) version 5.4. was used to analyze the relevant data using the Mantel–Haenszel method (12). I^2^ values were applied to estimate heterogeneity among the included clinical trials, which were classified into three grades: low, moderate, and high (I2 values <25%, 25%–50%, and >50%, respectively) (13). When I^2^ was greater than 50%, significant heterogeneity was considered, and the source of heterogeneity was determined by subgroup analysis. Owing to the inherent heterogeneity among the included trials, the random effect (RE) was applied to analyze the odds ratio (OR) and corresponding 95% confidence interval (CI) (14). Funnel plots derived from the fixed effect (FE) model were used to evaluate publication bias. All reported P values were two-sided, and P < 0.05 was deemed to be statistically significant.

## Results

### Literature search results

**Figure 1:**
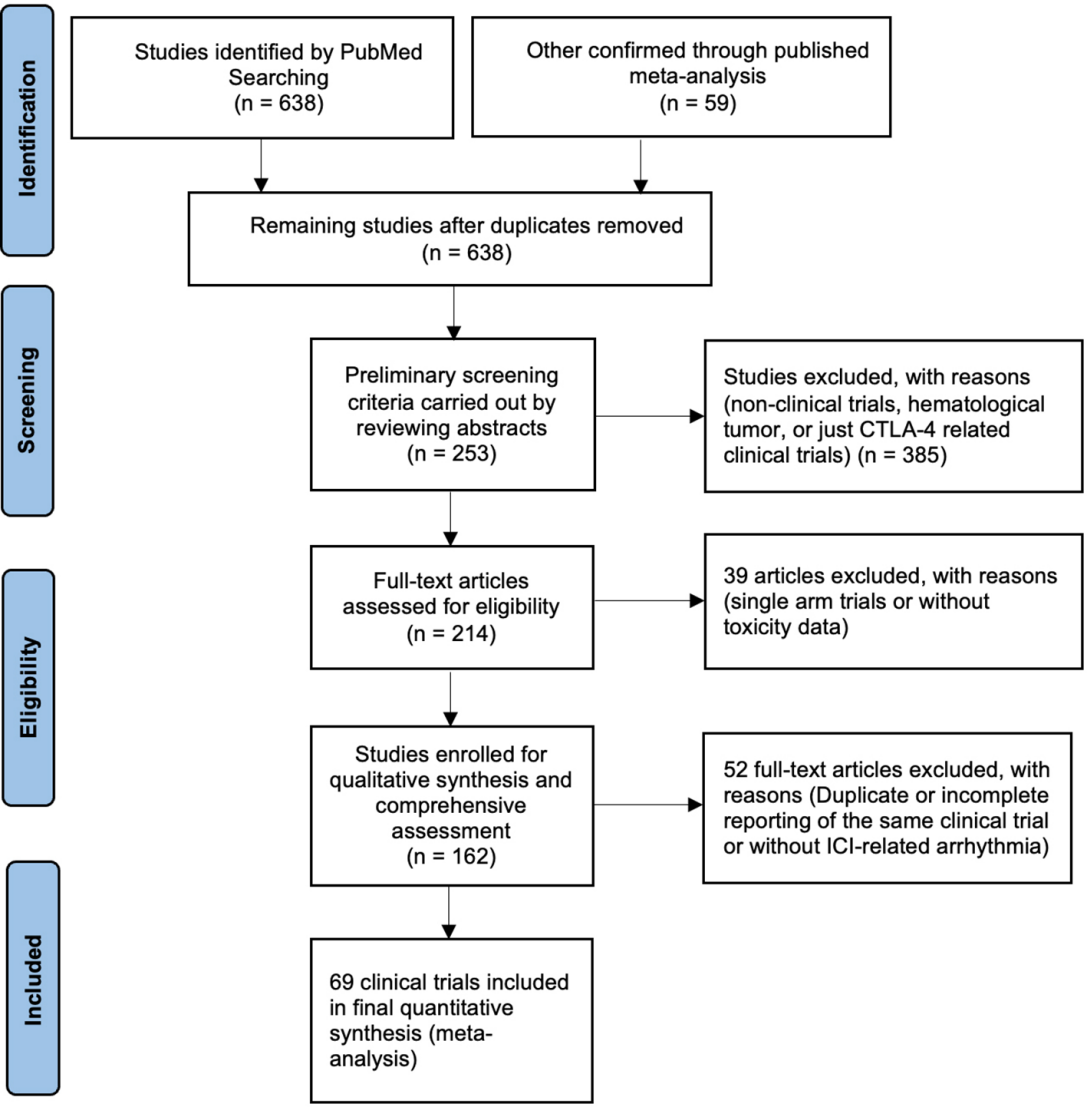
The flow diagram of the included randomized controlled trials (RCTs).

**Figure 2:**
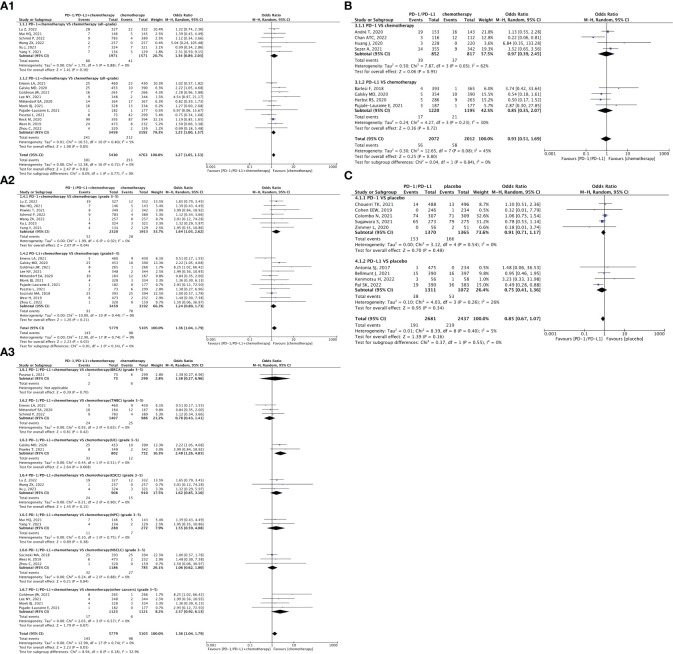
Forest plots depicting the risk of hypertension in PD-1/PD-L1 + chemotherapy versus chemotherapy. **(A1)** The risk of hypertension of all-grade: subgroup analyses were conducted according to PD-1/PD-L1. **(A2)** The risk of hypertension of grade 3-5: subgroup analyses were performed based on PD-1/PD-L1. **(A3)** The risk of hypertension of grade 3-5: subgroup analyses were performed based on types of tumors. Forest plot depicting the risk of hypertension in PD-1/PD-L1 versus chemotherapy. **(B)** The risk of hypertension of all-grade: subgroup analysis was conducted according to PD-1/PD-L1. Forest plot depicting the risk of hypertension in PD-1/PD-L1 versus placebo. **(C)** The risk of hypertension of all-grade: subgroup analysis was conducted according to PD-1/PD-L1.

We retrieved 638 relevant records from the PubMed database. The RCTs screening process was shown in **Figure 1**, and the baseline characteristics are presented in **Table 1**. Bias assessments of the included trials were completed and were presented in **Figure 2**. After thoroughly reviewing the complete texts of all trials included in this meta-analysis, a total of 10 prevalent cardiovascular toxicities were incorporated, comprising hypertension (n = 36) (22, 24, 25, 29–32, 34–37, 39, 40, 42–48, 51, 52, 54, 56, 62, 63, 65, 68, 69, 71, 72, 75, 77, 78, 81, 83, 84), hypotension (n = 14) (25, 29–32, 36, 40, 42, 52, 62, 68, 71, 75, 76, 78, 83, 84), arrhythmia (n = 32) (21–24, 29, 30, 32, 36, 37, 41, 42, 45–47, 57, 58, 61, 62, 65–69, 71, 72, 75, 76, 78, 83, 84), myocarditis (n = 31) (17, 21–25, 28, 30, 31, 33, 37, 38, 49, 50, 52, 53, 56, 59, 62, 63, 67, 68, 70, 72–74, 78–81, 84, 91), heart failure (n = 17) (20, 22, 25, 30–32, 34, 37, 45–47, 49, 62, 65, 67, 68, 78), myocardial infarction (n = 22) (15, 16, 23, 27, 30, 34, 36, 37, 39, 40, 46, 47, 52, 55, 62, 63, 65, 68, 70, 72, 78, 83, 84), pericardial diseases (n = 4) (32, 68, 76, 78), thrombosis (n = 18) (15, 25–27, 30, 34, 36, 40, 47, 52, 55, 62, 67, 68, 71, 76, 78, 83), embolism (n = 21) (15, 20, 22, 27, 30, 36, 38, 40–42, 45–48, 55, 62, 66–68, 83, 84), and vasculitis (n = 13) (19, 25, 27, 32, 51, 62, 64, 67, 68, 72, 80–84).

**Table 1:** The baseline characteristics of the RCTs included in this meta-analysis (Total of 69 clinical trials).

NO	First author and year	Study	Treatment lines	Tumor type	Drug	PD-1/ PD-L1	Treatment regimen	Enrollment
PD-1/PD-L1 + chemotherapy VS chemotherapy								
1	Forde PM, 2022 (15)	CheckMate 816 (NCT 02998528)	first line	NSCLC	Nivolumab	PD-1	Nivolumab + platinum-based chemotherapy VS platinum-based chemotherapy	352
2	Langer CJ, 2016 (16)	KEYNOTE-021 (NCT 02039674)	first line	NSCLC	Pembrolizumab	PD-1	Pembrolizumab + carboplatin VS carboplatin + pemetrexed	121
3	Rodríguez-Abreu D, 2021 (17)	KEYNOTE-189 (NCT 02578680)	first line	NSCLC	Pembrolizumab	PD-1	Pembrolizumab + pemetrexed-platinum VS pemetrexed-platinum	607
	Garassino MC, 2023 (18)							
4	Novello S, 2023 (19)	KEYNOTE-407 (NCT 02775435)	first line	NSCLC	Pembrolizumab	PD-1	Pembrolizumab + carboplatin+paclitaxel/nab-paclitaxel VS carboplatin + paclitaxel/nab-paclitaxel	558
5	Zhou C, 2021 (20)	CameL (NCT 03134872)	first line	NSCLC	Camrelizumab	PD-1	Camrelizumab + carboplatin + pemetrexed VS carboplatin + pemetrexed	412
6	Wang Z, 2023 (21)	CHOICE-01 (NCT 03856411)	first line	NSCLC	Toripalimab	PD-1	Toripalimab + nab-paclitaxel + carboplatin VS nab-paclitaxel + carboplatin	464
7	Lu Z, 2022 (22)	ORIENT-15(NCT 03748134)	first line	ESCC	Sintilimab	PD-1	Sintilimab + cisplatin + paclitaxel VS cisplatin + paclitaxel	659
8	Luo H, 2021 (23)	ESCORT-1st (NCT 03691090)	first line	ESCC	Camrelizumab	PD-1	Camrelizumab + paclitaxel + cisplatin VS paclitaxel + cisplatin	595
9	Wang ZX, 2022 (24)	JUPITER-06 (NCT 03829969)	first line	ESCC	Toripalimab	PD-1	Toripalimab + paclitaxel + cisplatin VS paclitaxel + cisplatin	514
10	Xu J, 2023 (25)	RATIONALE-306 (NCT 03783442)	first line	ESCC	Tislelizumab	PD-1	Tislelizumab + platinum agent and fluoropyrimidine/capecitabine/paclitaxel VS platinum agent and fluoropyrimidine/capecitabine/paclitaxel	645
11	Janjigian YY, 2021 (26)	CheckMate 649 (NCT 02872116)	first line	GJC	Nivolumab	PD-1	Nivolumab + capecitabine+oxaliplatin / leucovorin+fluorouracil+oxaliplatin VS capecitabine+oxaliplatin / leucovorin+fluorouracil+oxaliplatin	1549
12	Kang YK, 2022 (27)	CheckMate 649 (NCT 02872116)	first line	GC/GJC	Nivolumab	PD-1	Nivolumb + oxaliplatin + capecitabine VS oxaliplatin + capecitabin	717
13	Tolaney SM, 2020 (28)	NCT 03051659	second or others	BRCA	Pembrolizumab	PD-1	Pembrolizumab + eribulin VS eribulin	88
14	Schmid P, 2022 (29)	KEYNOTE-522 (NCT 03036488)	first line	TNBC	Pembrolizumab	PD-1	Pembrolizumab + paclitaxel + carboplatin VS paclitaxel + carboplatin	1172
15	Powles T, 2021 (30)	KEYNOTE-361 (NCT 02853305)	first line	UC	Pembrolizumab	PD-1	Pembrolizumab + gemcitabine+cisplatin/carboplatin VS gemcitabine+cisplatin/carboplatin	691
16	Mai HQ, 2021 (31)	JUPITER-02 (NCT 03581786)	first line	NPC	Toripalimab	PD-1	Toripalimab +gemcitabine-cisplatin VS gemcitabine-cisplatin	289
17	Yang Y, 2021 (32)	CAPTAIN-1st(NCT 03707509)	first line	NPC	Camrelizumab	PD-1	Camrelizumab + gemcitabine + cisplatin VS gemcitabine + cisplatin	263
18	Nishio M, 2021 (33)	IMpower132 (NCT 02657434)	first line	NSCLC	Atezolizumab	PD-L1	Atezolizumab + carboplatin / cisplatin and pemetrexed VS carboplatin / cisplatin and pemetrexed	565
19	Socinski MA, 2018 (34)	IMpower150 (NCT 02366143)	first line	NSCLC	Atezolizumab	PD-L1	Atezolizumab + bevacizumab + carboplatin + paclitaxel VS bevacizumab + carboplatin + paclitaxel	787
	Reck M, 2020 (35)							
20	West H, 2019 (36)	IMpower130 (NCT 02367781)	first line	NSCLC	Atezolizumab	PD-L1	Atezolizumab + carboplatin + nab-paclitaxel VS carboplatin + nab-paclitaxel	705
21	Zhou C, 2022 (37)	GEMSTONE-302 (NCT 03789604)	first line	NSCLC	Sugemalimab	PD-L1	Sugemalimab + platinum-based chemotherapy VS platinum-based chemotherapy	479
22	Johnson ML, 2023 (38)	POSEIDON (NCT 03164616)	first line	NSCLC	Durvalumab	PD-L1	Durvalumab + platinum-based chemotherapy VS platinum-based chemotherapy	667
23	Paz-Ares L, 2019 (39)	CASPIAN(NCT 03043872)	first line	SCLC	Durvalumab	PD-L1	Durvalumab + platinum–etoposide VS platinum–etoposide	531
	Goldman JW, 2021 (40)							
24	Wang J, 2022 (41)	CAPSTONE-1(NCT 03711305)	first line	SCLC	Adebrelimab	PD-L1	Adebrelimab + carboplatin + etoposide VS carboplatin + etoposide	462
25	Pusztai L, 2021 (42)	I-SPY2 (NCT 01042379)	first line	BRCA	Durvalumab	PD-L1	Durvalumab + olaparib + paclitaxel VS paclitaxel	372
26	Emens LA, 2021 (43)	IMpassion130 (NCT 02425891)	first line	TNBC	Atezolizumab	PD-L1	Atezolizumab + nab-paclitaxel VS nab-paclitaxel	890
27	Mittendorf EA, 2020 (44)	IMpassion031 (NCT 03197935)	first line	TNBC	Atezolizumab	PD-L1	Atezolizumab + nab-paclitaxel + doxorubicin + cyclophosphamide VS nab-paclitaxel + doxorubicin + cyclophosphamide	331
28	Pujade-Lauraine E, 2021 (45)	JAVELIN Ovarian 200(NCT 02580058)	first line	Multiple cancers	Avelumab	PD-L1	Avelumab + pegylated liposomal doxorubicin VS pegylated liposomal doxorubicin	359
29	Lee NY, 2021 (46)	JAVELIN Head and Neck 100(NCT 02952586)	first line	HNSCC	Avelumab	PD-L1	Avelumab+chemoradiotherapy VS chemoradiotherapy	692
30	Monk BJ, 2021 (47)	JAVELIN Ovarian 100(NCT 02718417)	first line	EOC	Avelumab	PD-L1	Avelumab + carboplatin + paclitaxel VS carboplatin + paclitaxel + observation	662
31	Moore KN, 2021 (48)	IMagyn050/GOG 3015/ENGOT-OV39(NCT 03038100)	first line	OC	Atezolizumab	PD-L1	Atezolizumab + bevacizumab + carboplatin + paclitaxel VS bevacizumab + carboplatin + paclitaxel	1286
32	Powles T, 2022 (49)	IMbassador 250 (NCT 03016312)	second or others	PCA	Atezolizumab	PD-L1	Atezolizumab + enzalutamide VS enzalutamide	750
33	Mettu NB, 2022 (50)	BACCI (NCT 02873195)	second or others	CRC	Atezolizumab	PD-L1	Atezolizumab + capecitabine + bevacizumab VS capecitabine + bevacizumab	132
34	Galsky MD, 2020 (51)	IMvigor130 (NCT 02807636)	first line	UC	Atezolizumab	PD-L1	Atezolizumab+platinum-based chemotherapy VS platinum-based chemotherapy	843
PD-1/PD-L1 VS chemotherapy								
1	Huang J, 2020 (52)	ESCORT (NCT 03099382)	second or others	ESCC	Camrelizumab	PD-1	Camrelizumab VS docetaxel/irinotecan	448
2	Kojima T, 2020 (53)	KEYNOTE-181 (NCT 02564263)	second or others	ESCC	Pembrolizumab	PD-1	Pembrolizumab VS paclitaxel/docetaxel/irinotecan	610
3	Chan ATC, 2023 (54)	KEYNOTE-122 (NCT 02611960)	second or others	NPC	Pembrolizumab	PD-1	Pembrolizumab VS capecitabine/gemcitabine/docetaxel	228
4	Diaz LA Jr, 2022 (55)	KEYNOTE-177 (NCT 02563002)	first line	CRC	Pembrolizumab	PD-1	Pembrolizumab VS 5-fluorouracil–based therapy	296
	André T, 2020 (56)							
5	Powles T, 2021 (30)	KEYNOTE-361 (NCT 02853305)	first line	UC	Pembrolizumab	PD-1	Pembrolizumab VS gemcitabine+cisplatin/carboplatin	644
6	Winer EP, 2021 (57)	KEYNOTE-119 (NCT 02555657)	second or others	TNBC	Pembrolizumab	PD-1	Pembrolizumab VS capecitabine/eribulin/gemcitabine/vinorelbine	601
7	Herbst RS, 2016 (58)	KEYNOTE-010 (NCT 01905657)	second or others	NSCLC	Pembrolizumab	PD-1	Pembrolizumab VS docetaxel	652
8	Mok TSK, 2019 (59)	KEYNOTE-042 (NCT 02220894)	first line	NSCLC	Pembrolizumab	PD-1	Pembrolizumab VS platinum-based chemotherapy	1251
	de Castro G Jr, 2023 (60)							
9	Borghaei H, 2015 (61)	CheckMate 057 (NCT 01673867)	second or others	NSCLC	Nivolumab	PD-1	Nivolumab VS docetaxel	555
10	Sezer A, 2021 (62)	EMPOWER-Lung 1 (NCT 03088540)	first line	NSCLC	Cemiplimab	PD-1	Cemiplimab VS platinum-doublet chemotherapy	697
11	Barlesi F, 2018 (63)	JAVELIN Lung 200 (NCT 02395172)	second or others	NSCLC	Avelumab	PD-L1	Avelumab VS docetaxel	758
12	Jassem J, 2021 (64)	IMpower110 (NCT 02409342)	first line	NSCLC	Atezolizumab	PD-L1	Atezolizumab VS platinum-based chemotherapy	549
	Herbst RS, 2020 (65)							
13	Galsky MD, 2020 (51)	IMvigor130 (NCT 02807636)	first line	UC	Atezolizumab	PD-L1	Atezolizumab VS platinum-based chemotherapy	744
14	van der Heijden MS, 2021 (66)	IMvigor211 (NCT 02302807)	second or others	UC	Atezolizumab	PD-L1	Atezolizumab VS vinflunine/paclitaxel/docetaxel	902
15	Powles T, 2020 (67)	DANUBE (NCT 02516241)	first line	UC	Durvalumab	PD-L1	Durvalumab VS gemcitabine+cisplatin/carboplatin	658
16	Pujade-Lauraine E, 2021 (45)	JAVELIN Ovarian 200(NCT 02580058)	first line	Multiple cancers	Avelumab	PD-L1	Avelumab VS pegylated liposomal doxorubicin	364
PD-1/PD-L1 VS placebo								
1	Choueiri TK, 2021 (68)	KEYNOTE-564 (NCT 03142334)	second or others	RCC	Pembrolizumab	PD-1	Pembrolizumab VS placebo	984
	Powles T, 2022 (69)							
2	Janjigian YY, 2021 (70)	KEYNOTE-811 (NCT 03615326)	second or others	GC	Pembrolizumab	PD-1	Pembrolizumab VS Placebo	433
3	Cohen EEW, 2019 (71)	KEYNOTE-040 (NCT 02252042)	second or others	HNSCC	Pembrolizumab	PD-1	Pembrolizumab VS Standard-of-Care	480
4	Colombo N, 2021 (72)	KEYNOTE-826 (NCT 03635567)	first line	CCA	Pembrolizumab	PD-1	Pembrolizumab VS Placebo	616
5	Eggermont AMM, 2020 (73)	KEYNOTE-054(NCT 02362594)	second or others	melanoma	Pembrolizumab	PD-1	Pembrolizumab VS Placebo	1011
6	Long GV, 2022 (74)	KEYNOTE-716 (NCT 03553836)	second or others	melanoma	Pembrolizumab	PD-1	Pembrolizumab VS Placebo	969
7	Zimmer L, 2020 (75)	IMMUNED (NCT 02523313)	second or others	melanoma	Nivolumab	PD-1	Nivolumab VS Placebo	107
8	Fennell DA, 2021 (76)	CONFIRM (NCT 03063450)	second or others	mesothelioma	Nivolumab	PD-1	Nivolumab VS placebo	332
9	Sugawara S, 2021 (77)	TASUKI-52 (NCT 03117049)	first line	NSCLC	Nivolumab	PD-1	Nivolumab VS Placebo	548
10	Antonia SJ, 2017 (78)	PACIFIC (NCT 02125461)	second or others	NSCLC	Durvalumab	PD-L1	Durvalumab VS Placebo	709
11	Zhou Q, 2022 (79)	GEMSTONE-301 (NCT 03728556)	second or others	NSCLC	Sugemalimab	PD-L1	Sugemalimab VS placebo	381
12	Felip E, 2021 (80)	IMpower010 (NCT 02486718)	second or others	NSCLC	Atezolizumab	PD-L1	Atezolizumab VS placebo	990
	Kenmotsu H, 2022 (81)							
13	Horn L, 2018 (82)	IMpower133 (NCT 02763579)	first line	SCLC	Atezolizumab	PD-L1	Atezolizumab VS Placebo	394
14	Bellmunt J, 2021 (83)	IMvigor010 (NCT 02450331)	first line	UC	Atezolizumab	PD-L1	Atezolizumab VS Observation	787
15	Pal SK, 2022 (84)	IMmotion010(NCT 03024996)	second or others	RCC	Atezolizumab	PD-L1	Atezolizumab VS placebo	773
PD-1/PD-L1 + CTLA-4 VS PD-1/PD-L1								
1	Antonia SJ, 2016 (85)	CheckMate 032 (NCT 01928394)	second or others	SCLC	Nivolumab	PD-1	Nivolumab + ipilimumab VS nivolumab	159
2	Boyer M, 2021 (86)	KEYNOTE-598 (NCT 03302234)	first line	NSCLC	Pembrolizumab	PD-1	Pembrolizumab+ipilimumab VS pembrolizumab	563
3	Gettinger SN, 2021 (87)	Lung-MAP S1400I(NCT 02785952)	second or others	SCLC	Nivolumab	PD-1	Nivolumab + ipilimumab VS nivolumab	247
4	Hodi FS, 2018 (88)	CheckMate 067 (NCT 01844505)	first line	melanoma	Nivolumab	PD-1	Nivolumab + ipilimumab VS Nivolumab	626
5	Powles T, 2020 (67)	DANUBE (NCT 02516241)	first line	UC	Durvalumab	PD-L1	Durvalumab + tremelimumab VS Durvalumab	685
PD-1/PD-L1 + CTLA-4 VS chemotherapy								
1	Baas P, 2021 (89)	CheckMate 743 (NCT 02899299)	first line	pleural mesothelioma	Nivolumab	PD-1	Nivolumab + ipilimumab VS chemotherapy	584
2	Paz-Ares L, 2021 (90)	CheckMate 9LA (NCT 03215706)	first line	NSCLC	Nivolumab	PD-1	Nivolumab + ipilimumab VS chemotherapy	707
3	Powles T, 2020 (67)	DANUBE (NCT 02516241)	first line	UC	Durvalumab	PD-L1	Durvalumab + tremelimumab VS Chemotherapy	653

PD-1, Programmed cell death 1; PD-L1, Programmed cell death 1 ligand 1; CTLA-4, anti-cytotoxic T-lymphocyte antigen-4; HR, Hazard Ratios; OR, Odds Ratio; CI, Confidence Interval; RE, Random Effect; FE, Fixed Effect; NSCLC, Non-Small-Cell Lung Cancer; SCLC, Small-Cell Lung Cancer; BRCA, Breast Cancer; UC, Urothelial Carcinoma; HNSCC, Head and Neck Squamous Cell Carcinoma; CCA, Cervical Cancer; TNBC, Triple-Negative Breast Cancer; GC, Gastric Cancer; GC/GJC, Gastric or gastro-oesophageal junction cancer; ESCC, Oesophagea/Esophagea Squamous Cell Carcinoma; NPC, Nasopharyngeal Cancer; CRC, Colorectal Cancer; EOC, Epithelial Ovarian Cancer; OC, Ovarian Cancer; GEC, Gastroesophageal adenocarcinoma; RCC, Renal Cell Carcinoma; PCA, Prostate Cancer; HCC, Hepatocellular Carcinoma; EC, Esophageal Cancer; MPM, Malignant Pleural Mesothelioma.

### Characteristics of identified trials

We first divided the 63 clinical trials into five groups according to treatment regimen. The specific grouping methods are as follows.

Group 1: PD-1/PD-L1 + chemotherapy versus chemotherapy; n = 34 (15, 16, 19–51, 91). Seventeen clinical trials included PD-1 (15–17, 19–32) and seventeen clinical trials included PD-L1 (33–51).
Group 2: PD-1/PD-L1 versus chemotherapy; n = 16 (30, 45, 51–67). Ten clinical trials included PD-1 (30, 52–62) and six included PD-L1 (45, 51, 63–67).
Group 3: PD-1/PD-L1 versus placebo; n = 15 (17, 27, 68–82, 84). Nine clinical trials included PD-1 (68–77) and six included PD-L1 (78–84).
Group 4: PD-1/PD-L1 + CTLA-4 versus PD-1/PD-L1; n = 5 (67, 85–88). Four clinical trials included PD-1 (85–88) and one included PD-L1 (67).
Group 5: PD-1/PD-L1 + CTLA-4 versus chemotherapy; n = 3 (67, 89, 90). Two clinical trials included PD-1 (89, 90) and one included PD-L1 (67).

### Risk of hypertension

Thirty-six clinical trials reported hypertension (22, 24, 25, 29–32, 34–37, 39, 40, 42–48, 51, 52, 54, 56, 62, 63, 65, 68, 69, 71, 72, 75, 77, 78, 81, 83, 84). In comparison to chemotherapy, PD-1/PD-L1 + chemotherapy resulted in a significantly increased risk of all-grade hypertension (OR = 1.27, 95% CI [1.05, 1.53], p = 0.01, I^2 = ^0%; **Figure 2A1**), especially for the subgroup of first-line treatment (OR = 1.27, 95% CI [1.05, 1.53], p = 0.01, I^2 = ^0%; **Figure 2A1**) (22, 24, 25, 29, 31, 32, 35–37, 40, 42–47, 51). Similar trend were also be found in grade 3–5 hypertension (OR = 1.36, 95% CI [1.04, 1.79], p = 0.03, I^2 = ^0%; **Figure 2A2**). Among them, the PD-1 subgroup (OR = 1.64, 95% CI [1.03, 2.62], p = 0.04, I^2 = ^0%; **Figure 2A2**), first-line treatment (OR = 1.36, 95% CI [1.04. 1.79], p = 0.03, I^2 = ^0%; **Figure 2A2**), or urothelial carcinoma (UC) (OR = 2.48, 95% CI [1.26, 4.85], p = 0.008, I^2 = ^0%; **Figure 2A3**) were more likely to cause grade 3–5 hypertension (22, 24, 25, 29–32, 34, 36, 37, 40, 42–47, 51). No heterogeneity was observed among the studies.

Compared with chemotherapy alone (**Figure 2B**) (45, 51, 52, 54, 56, 62, 63, 65) or the placebo (**Figure 2C**) (68, 71, 72, 75, 77), the effects of PD-1/PD-L1 inhibitors on hypertension, indicated by non-significant statistical analysis results, were weaker than those of the control groups. The corresponding funnel plots are shown in the **Supplementary Data** (**Supplementary Figure 2**).

### Risk of hypotension

**Figure 3:**
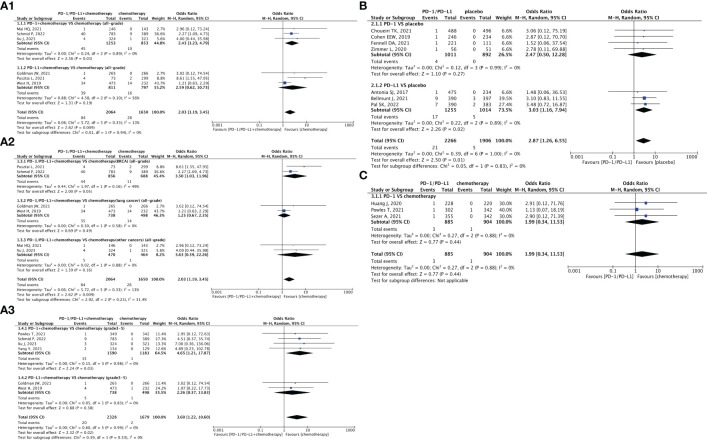
Forest plots depicting the risk of hypotension in PD-1/PD-L1 + chemotherapy versus chemotherapy. **(A1)** The risk of hypotension of all-grade: subgroup analyses were conducted according to PD-1/PD-L1. **(A2)** The risk of hypotension of all-grade: subgroup analyses were conducted according to types of tumors. **(A3)** The risk of hypotension of grade 3-5: subgroup analysis was conducted according to PD-1/PD-L1. Forest plot depicting the risk of hypotension in PD-1/PD-L1 versus placebo. **(B)** The risk of hypotension of all-grade: subgroup analysis was conducted according to PD-1/PD-L1. Forest plot depicting the risk of hypotension in PD-1/PD-L1 versus chemotherapy. **(C)** The risk of hypotension of grade 3-5: subgroup analysis was conducted according to PD-1.

There were fourteen clinical trials reporting hypotension (25, 29–32, 36, 40, 42, 52, 62, 68, 71, 75, 76, 78, 83, 84). The risk of all-grade hypotension (OR = 2.03, 95% CI [1.19, 3.45], p = 0.009, I^2 = ^13%; **Figure 3A1**) and grade 3–5 hypotension (OR = 3.60, 95% CI [1.22, 10.60], p = 0.02, I^2 = ^0%; **Figure 3A3**) associated with chemotherapy were significantly lower than those associated with PD-1/PD-L1 + chemotherapy. This difference was particularly notable in the PD-1 subgroup [(all-grade (OR = 2.43, 95% CI [1.23, 4.79], p = 0.01, I^2 = ^0%; **Figure 3A1**); grade 3–5 (OR = 4.65, 95% CI [1.21, 17.87], p = 0.03, I^2 = ^0%; **Figure 3A3**], and first-line treatment subgroup [all-grade (OR = 2.03, 95% CI [1.19. 3.45], p = 0.009, I^2 = ^13%; **Figure 3A1**); grade 3–5 (OR = 3.60, 95% CI [1.22, 10.60], p = 0.02, I^2 = ^0%; **Figure 3A3**)] (25, 29, 31, 36, 40, 42). Furthermore, in the subgroup of breast cancer (BRCA), PD-1/PD-L1 + chemotherapy exhibited a tendency toward a higher risk of all-grade hypotension (OR = 3.50, 95% CI [1.03, 11.96], p = 0.05, I^2 = ^49%; **Figure 3A2**).

Compared to placebo, PD-1/PD-L1 substantially increased the risk of all-grade hypotension (OR = 2.87, 95% CI [1.26, 6.55], p = 0.01, I^2 = ^0%; **Figure 3B**), especially PD-L1 (OR = 3.03, 95% CI [1.16, 7.94], p = 0.02, I^2 = ^0%; **Figure 3B**) (68, 71, 75, 76, 78, 83, 84). No significant heterogeneity was observed in the aforementioned results. PD-1/PD-L1 did not demonstrate a higher risk of grade 3–5 hypotension when compared to chemotherapy alone (**Figure 3C**) (30, 52, 62). The corresponding funnel plots are shown in **Supplementary Data** (**Supplementary Figure 3**).

### Risk of arrhythmia

**Figure 4:**
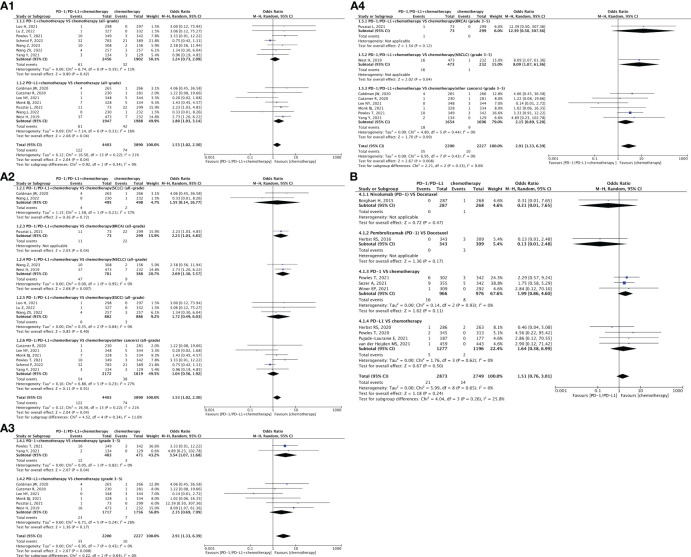
Forest plots depicting the risk of arrhythmia in PD-1/PD-L1 + chemotherapy versus chemotherapy. **(A1)** The risk of arrhythmia of all-grade: subgroup analyses were conducted according to PD-1/PD-L1. **(A2)** The risk of arrhythmia of all-grade: subgroup analyses were conducted according to types of tumors. **(A3)** The risk of arrhythmia of grade 3-5: subgroup analyses were conducted according to PD-1/PD-L1. **(A4)** The risk of arrhythmia of grade 3-5: subgroup analyses were conducted according to types of tumors. Forest plot depicting the risk of arrhythmia in PD-1/PD-L1 versus chemotherapy. **(B)** The risk of arrhythmia of all-grade: subgroup analysis was conducted according to PD-1/PD-L1.

Thirty-two clinical trials reported arrhythmia (21–24, 29, 30, 32, 36, 37, 41, 42, 45–47, 57, 58, 61, 62, 65–69, 71, 72, 75, 76, 78, 83, 84). Compared with chemotherapy, the combination of PD-1/PD-L1 inhibitors with chemotherapy exhibited a significantly higher risk of all-grade arrhythmia (OR = 1.53, 95% CI [1.02, 2.30], p = 0.04, I^2 = ^21%; **Figure 4A1**) and grade 3–5 arrhythmia (OR = 2.91, 95% CI [1.33, 6.39], p = 0.008, I^2 = ^0%; **Figure 4A3**). This effect was particularly prominent in the subgroups of first-line treatment [all-grade (OR = 1.53, 95% CI [1.02, 2.30], p = 0.04, I^2 = ^21%; **Figure 4A1**); grade 3–5 (OR = 2.91, 95% CI [1.33, 6.39], p = 0.008, I^2 = ^0%; **Figure 4A3**)], and non-small cell lung cancer (NSCLC) [all-grade (OR = 2.69, 95% CI [1.30, 5.57], p = 0.007, I^2 = ^0%; **Figure 4A2**); grade 3–5 (OR = 8.09, 95% CI [1.07, 61.36], p = 0.04; **Figure 4A4**)] (21–24, 29, 30, 32, 36, 40–42, 46, 47). Specifically, the combination of PD-L1 and chemotherapy demonstrated a higher risk of causing all-grade arrhythmias (OR = 1.80, 95% CI [1.03, 3.14], p = 0.04, I^2 = ^16%; **Figure 4A1**), whereas PD-1 combined with chemotherapy was more prone to inducing grade 3–5 arrhythmia (OR = 3.54, 95% CI [1.07, 11.68], p = 0.04, I^2 = ^0%; **Figure 4A3**). Additionally, among BRCA patients, there was an increased risk of developing all-grade arrhythmia with PD-1/PD-L1 + chemotherapy (OR = 2.23, 95% CI [1.03, 4.85], p = 0.04; **Figure 4A2**). Notably, no significant heterogeneity was observed among the findings.

**Figure 5:**
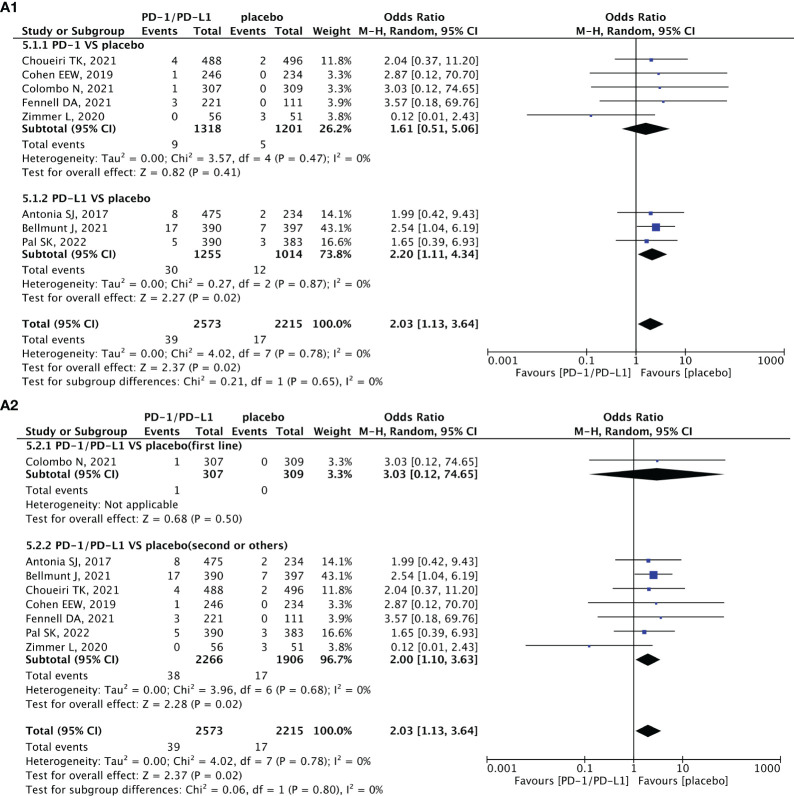
Forest plots depicting the risk of arrhythmia in PD-1/PD-L1 versus placebo. **(A1)** The risk of arrhythmia of all-grade: subgroup analyses were conducted according to PD-1/PD-L1. **(A2)** The risk of arrhythmia of all-grade: subgroup analyses were conducted according to treatment lines.

When comparing PD-1/PD-L1 inhibitors (nivolumab and pembrolizumab) with chemotherapy (specifically docetaxel), it was observed that nivolumab and pembrolizumab carried a lower risk of inducing hypotension; however, the difference was not statistically significant (**Figure 4B**) (30, 45, 57, 58, 61, 62, 65–67). Compared to placebo, PD-1/PD-L1 inhibitors showed a tendency toward a higher risk of all-grade arrhythmia (OR = 2.03, 95% CI [1.13, 3.64], p = 0.02, I^2 = ^0%; **Figure 5A**), particularly within the PD-L1 subgroup (OR = 2.20, 95% CI [1.11, 4.34], p = 0.02, I^2 = ^0%; **Figure 5A1**) and second-line treatment subgroup (OR = 2.00, 95% CI [1.10, 3.63], p = 0.02, I^2 = ^0%; **Figure 5A2**) (68, 71, 72, 75, 76, 78, 83, 84). No heterogeneity was observed in the aforementioned results. The corresponding funnel plots are presented in **Supplementary Data** (**Supplementary Figures 4**, **5**).

### Risk of myocarditis

**Figure 6:**
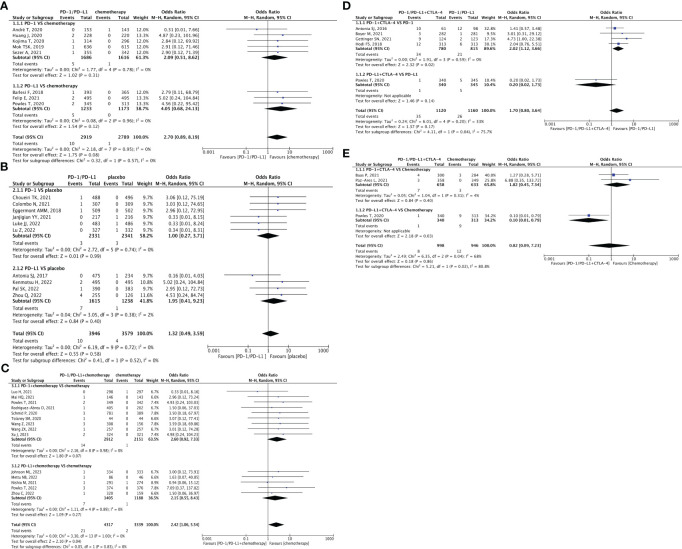
Forest plot depicting the risk of myocarditis in PD-1/PD-L1 versus chemotherapy. **(A)** The risk of myocarditis of all-grade: subgroup analysis was conducted according to PD-1/PD-L1. Forest plot depicting the risk of myocarditis in PD-1/PD-L1 versus placebo. **(B)** The risk of myocarditis of all-grade: subgroup analysis was conducted according to PD-1/PD-L1. Forest plot depicting the risk of myocarditis in PD-1/PD-L1 + chemotherapy versus chemotherapy. **(C)** The risk of myocarditis of all-grade: subgroup analysis was conducted according to PD-1/PD-L1. Forest plot depicting the risk of cardiovascular toxicities in PD-1/PD-L1 + CTLA-4 versus PD-1/PD-L1. **(D)** The risk of cardiovascular toxicities of all-grade: subgroup analysis was conducted according to PD-1/PD-L1. Forest plot depicting the risk of cardiovascular toxicities in PD-1/PD-L1 + CTLA-4 versus chemotherapy. **(E)** The risk of cardiovascular toxicities of all-grade: subgroup analysis was conducted according to PD-1/PD-L1.

The adverse effects of myocarditis were reported in thirty-one clinical trials (17, 21–25, 28, 30, 31, 33, 37, 38, 49, 50, 52, 53, 56, 59, 62, 63, 67, 68, 70, 72–74, 78–81, 84, 91). No significant difference was observed in the risk of myocarditis between PD-1/PD-L1 monotherapy and chemotherapy (**Figure 6A**) (52, 53, 56, 59, 62, 63, 67, 80) or between PD-1/PD-L1 monotherapy and placebo (**Figure 6B**) (22, 68, 70, 72–74). However, the risk of all-grade myocarditis associated with chemotherapy was significantly lower than that associated with PD-1/PD-L1 + chemotherapy (OR = 2.42, 95% CI [1.06, 5.54], p = 0.04, I^2 = ^0%; **Figure 6C**) (17, 21–25, 28, 30, 31, 33, 37, 38, 50, 69, 91). No heterogeneity was found in the above result. The corresponding funnel plots are provided in the **Supplementary Data** (**Supplementary Figures 6A–C**).

### Risk of cardiovascular toxicity associated with CTLA-4

Five clinical trials compared PD-1/PD-L1 + CTLA-4 with PD-1/PD-L1 (67, 85–88). Among them, four RCTs included PD-1, and the results suggested a significantly higher risk following combination therapy than following PD-1 monotherapy (OR = 2.02, 95% CI [1.12, 3.66], p = 0.02, I^2 = ^0%; **Figure 6D**). Three clinical trials compared PD-1/PD-L1 + CTLA-4 versus chemotherapy (67, 89, 90). Only one of these studies involved PD-L1 combined with CTLA-4, and the results indicated a lower risk of cardiovascular toxicity for this treatment than chemotherapy (OR = 0.10, 95% CI [0.01, 0.79], p = 0.03; **Figure 6E**). The corresponding funnel plots are provided in the **Supplementary Data** (**Supplementary Figure 6D, E**).

### Risk of myocardial infarction, heart failure, and pericardial diseases

There were twenty-two clinical trials reporting on myocardial infarction (15, 16, 23, 27, 30, 34, 36, 37, 39, 40, 46, 47, 52, 55, 62, 63, 65, 68, 70, 72, 78, 83, 84). Heart failure was reported in seventeen clinical trials (20, 22, 25, 30–32, 34, 37, 45–47, 49, 62, 65, 67, 68, 78). Only four clinical trials reported pericardial diseases (32, 68, 76, 78). No statistically significant differences were observed in the risk of all-grade heart failure between the PD-1/PD-L1 versus chemotherapy or PD-1/PD-L1 + chemotherapy versus chemotherapy groups (20, 22, 25, 31, 32, 34, 37, 45–47, 49, 62, 63, 65, 67), myocardial infarction (15, 16, 23, 27, 30, 34, 36, 37, 39, 40, 46, 47, 52, 55, 62, 63, 65), or pericardial diseases (32, 68, 76, 78). Additionally, no statistically significant difference was observed in the risk of all-grade heart failure (78, 84) or myocardial infarction (68, 70, 72, 78, 83, 84) with PD-1/PD-L1 or placebo. The specific statistical data is presented in **Tables 2**, **3**.

**Table 2:** The risk of all-grade myocardial infarction, heart failure, pericardial diseases, embolism, thrombosis and vasculis: subgroup analyses were carried out based on PD-1/PD-L1.

Treatment regimen		PD-1/PD-L1+chemotherapy VS chemotherapy	PD-1/PD-L1 VS chemotherapy	PD-1/PD-L1 VS placebo
**myocardial infraction**	PD-1	OR=0.69, 95% CI [0.11, 4.40], p=0.70	OR=0.80, 95% CI [0.20, 3.29], p=0.76	OR=2.16, 95% CI [0.46, 10.09], p=0.33
	PD-L1	OR=0.86, 95% CI [0.32, 2.32], p=0.77	OR=0.92, 95% CI [0.10, 8.91], p=0.95	OR=1.91, 95% CI [0.32, 11.36], p=0.48
**heart failure**	PD-1	OR=1.43, 95% CI [0.33, 6.26], p=0.64	OR=0.72, 95% CI [0.16, 3.24], p=0.67	OR=2.04, 95% CI [0.18, 22.54], p=0.56
	PD-L1	OR=1.17, 95% CI [0.52, 2.63], p=0.70	OR=0.56, 95% CI [0.13, 2.30], p=0.42	OR=3.22, 95% CI [0.37, 28.43], p=0.29
**pericardial diseases**	PD-1	OR=0.96, 95% CI [0.06, 15.55], p=0.98	N/A	OR=3.82, 95% CI [0.44, 33.23], p=0.22
	PD-L1	OR=2.42, 95% CI [0.46, 12.82], p=0.03	N/A	OR=2.48, 95% CI [0.12, 51.79], p=0.56
**emobolism**	PD-1	OR=1.17, 95% CI [0.33, 4.13], p=0.81	OR=1.28, 95% CI [0.15, 10.61], p=0.82	OR=1.37, 95% CI [0.09, 19.88], p=0.82
	PD-L1	OR=1.05, 95% CI [0.66, 1.66], p=0.85	OR=1.49, 95% CI [0.18, 12.17], p=0.71	OR=1.03, 95% CI [0.26, 4.01], p=0.97
**thrombosis**	PD-1	OR=0.67, 95% CI [0.15, 2.98], p=0.60	OR=0.96, 95% CI [0.29, 3.15], p=0.95	OR=0.54, 95% CI [0.09, 3.47], p=0.52
	PD-L1	OR=1.74, 95% CI [0.79, 3.84], p=0.17	OR=0.18, 95% CI [0.01, 3.77], p=0.27	OR=0.58, 95% CI [0.12, 2.73], p=0.49
**vasculitis**	PD-1	OR=0.80, 95% CI [0.20, 3.29], p=0.76	OR=0.32, 95% CI [0.01, 7.89], p=0.49	OR=5.07, 95% CI [0.24, 105.95], p=0.30
	PD-L1	OR=0.80, 95% CI [0.20, 3.29], p=0.76	OR=0.83, 95% CI [0.17, 4.01], p=0.81	OR=1.02, 95% CI [0.24, 4.43], p=0.98

PD-1, Programmed cell death 1; PD-L1, Programmed cell death 1 ligand 1; OR, Odds Ratio; CI, Confidence Interval; N/A, not available.

**Table 3:** The risk of all-grade myocardial infarction, heart failure, pericardial diseases, embolism, thrombosis and vasculis: subgroup analyses were carried out based on treatment lines.

Treatment regimen		PD-1/PD-L1+chemotherapy VS chemotherapy	PD-1/PD-L1 VS chemotherapy	PD-1/PD-L1 VS placebo
**myocardial infraction**	first line	OR=0.82, 95% CI [0.34, 1.96], p=0.65	OR=1.22, 95% CI [0.30, 4.98], p=0.78	OR=1.62, 95% CI [0.20, 13.22], p=0.65
	second or others	N/A	OR=0.31, 95% CI [0.03, 3.03], p=0.32	OR=2.28, 95% CI [0.56, 9.25], p=0.25
**heart failure**	first line	OR=1.08, 95% CI [0.52, 2.25], p=0.84	OR=0.48, 95% CI [0.16, 1.45], p=0.19	N/A
	second or others	OR=4.05, 95% CI [0.45, 36.44], p=0.21	OR=4.67, 95% CI [0.22, 97.56], p=0.32	OR=2.62, 95% CI [0.52, 13.16], p=0.24
**pericardial diseases**	first line	OR=1.90, 95% CI [0.45, 7.93], p=0.38	N/A	N/A
	second or others	N/A	N/A	OR=3.30, 95% CI [0.57, 19.25], p=0.18
**emobolism**	first line	OR=1.06, 95% CI [0.69, 1.64], p=0.79	OR=1.21, 95% CI [0.26, 5.65], p=0.81	OR=0.33, 95% CI [0.01, 8.24], p=0.50
	second or others	N/A	OR=2.90, 95% CI [0.12, 71.42], p=0.51	OR=1.34, 95% CI [0.39, 4.65], p=0.64
**thrombosis**	first line	OR=1.41, 95% CI [0.70, 2.83], p=0.34	OR=0.64, 95% CI [0.20, 2.09], p=0.46	OR=0.54, 95% CI [0.09, 3.47], p=0.52
	second or others	N/A	OR=2.91, 95% CI [0.12, 71.76], p=0.51	OR=0.58, 95% CI [0.12, 2.73], p=0.49
**vasculitis**	first line	OR=1.51, 95% CI [0.86, 2.65], p=0.15	OR=0.82, 95% CI [0.17, 3.97], p=0.80	OR=1.35, 95% CI [0.09, 19.84], p=0.82
	second or others	N/A	OR=0.33, 95% CI [0.01, 8.19], p=0.50	OR=1.38, 95% CI [0.27, 7.19], p=0.70

PD-1: Programmed cell death 1; PD-L1: Programmed cell death 1 ligand 1; OR: Odds Ratio; CI: Confidence Interval; N/A, not available.

### Risk of embolism, thrombosis, and vasculitis

Twenty-one clinical trials reported embolism (15, 20, 22, 27, 30, 36, 38, 40–42, 45–48, 55, 62, 66–68, 83, 84), eighteen reported thrombosis (15, 25–27, 30, 34, 36, 40, 47, 52, 55, 62, 67, 68, 71, 76, 78, 83) and thirteen reported vasculitis (19, 25, 27, 32, 51, 62, 64, 67, 68, 72, 80–84). No significant differences were observed in the risk of all-grade embolism between the PD-1/PD-L1 versus chemotherapy/placebo group and the PD-1/PD-L1 + chemotherapy versus chemotherapy group (15, 20, 22, 27, 30, 36, 38, 40–42, 45–48, 55, 62, 66–68, 83, 84), thrombosis (15, 25–27, 30, 34, 36, 40, 47, 52, 55, 62, 67, 68, 71, 76, 78, 83), or vasculitis (19, 25, 27, 32, 51, 62, 64, 67, 68, 72, 80–84). The specific statistical data is presented in **Tables 2**, **3**.

## Discussion

This meta-analysis included recently completed RCTs and provided updated information on the cardiotoxicity of PD-1/PD-L1 inhibitors. With a larger sample size and more detailed subgroups, this study provided several novel findings, indicating that the combination of PD-1/PD-L1 inhibitors with chemotherapy carries a considerably higher risk of myocarditis and hypotension than conventional chemotherapy alone. An increasing number of people are now paying attention to the cardiovascular toxicities of PD-1/PD-L1, and this study provides strong supporting evidence for these concerns. Additionally, it assists doctors in making preliminary assessments of the potential causes of these side effects when they detect cardiovascular issues in patients. This, in turn, allows for a more significant improvement in patient prognosis without compromising their anti-tumor treatment. Additionally, this study supports previous meta-analyses (7, 8) and preclinical evidence (9) (92, 93), highlighting the substantial increase in cardiovascular toxicities associated with PD-1/PD-L1 inhibitors. Flow cytometry and metabolomic assays revealed that PD-1/PD-L1 treatment in mice resulted in an increase in the overall lymphocyte count and changes in lipid metabolism within the cardiac tissue. These findings provide evidence that PD-1/PD-L1 disrupts immune homeostasis and energy production in the heart (9). Furthermore, single-cell sequencing revealed that endothelial cells constituted the majority of cells in the cardiac interstitium. Notably, these endothelial cells, along with cardiomyocytes and vascular endothelial cells, exhibit high levels of PD-L1 expression on their surfaces (92, 93). The use of PD-1/PD-L1 inhibitors can enable T cells to nonselectively target normal cells in the heart. Consequently, these factors increase the risk of cardiovascular toxicity.

This study demonstrated a notable increase in the risk of hypertension with the use of PD-1/PD-L1 inhibitors in combination with chemotherapy (22, 24, 25, 29, 31, 32, 35–37, 40, 42–47, 51). This trend was specifically observed in the subgroups of PD-1 inhibitors, first-line treatment, and urothelial carcinoma (UC), which has not been reported in previous meta-analyses. This phenomenon may be attributed to the immune-enhancing effects of PD-1/PD-L1 inhibitors. Owing to the high expression of PD-L1 on vascular endothelial cells (94), medications that enhance non-specific attack by T cells can also cause damage to vascular endothelial cells. This weakens the ability of cells to regulate blood pressure, leading to blood pressure fluctuations (95). However, the exact mechanism requires further investigation. In addition, while PD-1/PD-L1 did not exhibit statistically significant outcomes compared with chemotherapy or placebo, it can be inferred that PD-1/PD-L1 carries a reduced risk of inducing hypertension compared with the placebo group. This novel fact should be applied in clinical settings; when hypertension occurs after using PD-1/PD-L1, initial focus should be on identifying factors unrelated to this medication, such as potential drug interactions, unhealthy lifestyle choices, underlying health conditions, age, or gender.

Despite the lack of significant differences in the risk of heart failure among the treatment regimens in this study (20, 22, 25, 31, 32, 34, 37, 45–47, 49, 62, 63, 65, 67, 78, 84), the potential detrimental effects of PD-1/PD-L1 on cardiac function should not be overlooked. Michel et al. (9) observed that six of seven patients with stage IV progressive melanoma treated with PD-1 had decreased left ventricular ejection fraction (LVEF) and exhibited no significant signs of myocarditis four weeks after the first treatment. In addition, this study also concluded that PD-1/PD-L1 alone (68, 71, 75, 76, 78, 83, 84) or in combination with chemotherapy (25, 29, 31, 36, 40, 42) leads to an appreciably higher risk of hypotension, which was first reported in a meta-analysis, and could not be ruled out as a manifestation of reduced ejection following a decrease in cardiac function due to PD-1/PD-L1. This trend was particularly evident in the PD-1 + chemotherapy, PD-L1 alone, first-line treatment, or breast cancer subgroups. In addition to diminished cardiac pumping, hypotension cannot exclude the less common drug-induced hypersensitivity syndrome (DIHS), which results from excessive activation of T-cell function by immune checkpoint inhibitors (ICIs) (96). Vasodilation and increased permeability of the vessel wall lead to plasma extravasation, which reduces the intravascular blood volume and vasogenic hypotension. However, the exact mechanisms remain to be further elucidated.

In a comparison of PD-1/PD-L1 + chemotherapy versus chemotherapy (21–24, 29, 30, 32, 36, 40–42, 46, 47) and PD-1/PD-L1 versus placebo (68, 71, 72, 75, 76, 78, 83, 84), the use of PD-1/PD-L1-related therapy was associated with a considerably increased risk of arrhythmias. Particularly in the NSCLC subgroup, the combination of PD-1/PD-L1 inhibitors with chemotherapy led to a notably higher occurrence of all-grade or grade 3–5 arrhythmia (21, 36). This is broadly consistent with the results of previous meta-analyses or reviews by Herrmann and Liu et al. (7, 97). In addition, although there was no statistically significant difference in the risk of arrhythmia between PD-1/PD-L1 inhibitors and chemotherapy, the two PD-1 inhibitors, nivolumab and pembrolizumab, exhibited a lower risk of arrhythmia than docetaxel. Thus, more important with docetaxel is the prevention of several serious complications, such as myocardial ischemia due to abnormal heart rhythms. Additionally, positive results may be obtained concerning the apparent subjective discomfort experienced by the patients. Currently, physicians can easily ascertain abnormal heart rhythms and collect these data using Holter (24h dynamic electrocardiogram) or other devices. However, additional fundamental research is required to investigate the mechanisms by which PD-1/PD-L1 affects the cardiac conduction system.

Clinical evidence has indicated that immunotherapy can cause myocarditis, which should be taken seriously. The severity of immune-associated myocarditis varies from mild cases without apparent inflammation to severe cases that may be associated with heart failure, cardiogenic shock, and a high mortality rate in the case of rapidly progressing fulminant myocarditis (98, 99). Hu et al. concluded that immunotherapy drastically increased the risk of myocardial disease compared with conventional antitumor therapy (100). This is the first study to provide evidence that the combination of PD-1/PD-L1 inhibitors and chemotherapy is associated with an elevated risk of myocarditis (17, 21–25, 28, 30, 31, 33, 37, 38, 50, 69, 91). However, no positive results were obtained in the subgroup analysis, which should be conducted in additional RCTs. The exact mechanism of immune-associated myocarditis remains unclear, but some preclinical studies have made some conjectures, such as inflammation due to T-cell activation (101). Given the poor prognosis of this disease, more clinical data and basic research are required.

The combination of PD-1/PD-L1 and CTLA-4 blockade substantially enhances the immune responses and survival rates in certain cancers (102). However, it also increases the risk of adverse effects. This study found that the risk of cardiovascular toxicity following PD-1 combined with CTLA-4 treatment was noticeably higher than following PD-1 treatment alone, and these results were consistent with prior findings. Preclinical trials have revealed that when PD-1 on the surface of myocardial cells binds to PD-L1 on the surface of T lymphocytes, it prevents T lymphocytes from attacking the myocardium. CTLA-4, on the other hand, prevents lymphocyte proliferation and spread. Therefore, the simultaneous inhibition of both pathways inevitably leads to indiscriminate T lymphocyte attacks on myocardial tissue, resulting in an increased risk of cardiovascular toxicity with the combined use of ICIs (103). Further research is required to decrease the occurrence of adverse event while maintaining the efficacy of the combination.

Cardiovascular toxicities associated with ICIs can be indicated by several biomarkers, including inflammatory markers such as C-reactive protein, erythrocyte sedimentation rate, and white blood cell count, as well as cardiac injury markers like troponin I, creatine kinase-MB, and brain natriuretic peptide. The development of ICI adverse effects is attributed to excessive enhancement of immune function, leading to inadvertent harm to normal cells. In response, we initially administered symptomatic treatments involving a variety of immunosuppressive agents, including corticosteroids, cytotoxic drugs, calcineurin inhibitors, and biologics. Secondly, the severity of the adverse effects needs to be assessed to determine whether temporary or permanent discontinuation of the medication is warranted. In addition, screening specific patients before initiating treatment can help prevent adverse effects. For instance, it is not recommended for individuals with autoimmune diseases, organ transplant recipients, patients with active hepatitis, or elderly patients to use ICIs. Furthermore, patients with pre-existing cardiovascular disorders should be monitored (104).

This meta-analysis further refined the cardiovascular toxicity of PD-1/PD-L1 through a comprehensive analysis of 69 RCTs. Moreover, there was no heterogeneity or insignificant heterogeneity among the RCTs included in this meta-analysis; thus, the results were reliable. However, this study had some limitations. Only 11% of the original studies searched reported the above cardiovascular toxicity events. In an initial comparison of morbidity data, PD-1/PD-L1 treatment resulted in a higher number of cardiovascular adverse events than conventional treatment. However, the final meta-analysis did not yield positive results. First, it can be inferred that PD-1/PD-L1 therapy is safe. However, it should also be noted that cardiovascular adverse events may not have received sufficient attention from doctors and patients, resulting in patients not seeking medical treatment promptly or first consulting physicians not collecting data on time. Therefore, due to the lack of sufficient sample size, this study was unable to collect baseline information for subgroup analyses of additional possible risk factors or to shed light on the specifics of chemotherapy. Furthermore, this meta-analysis exclusively included RCTs; most of these only reported a greater than certain percentage of cardiovascular toxicities, which may lead to the underreporting of some rare diseases with low incidence.

## Conclusion

The combination of PD-1/PD-L1 with chemotherapy increases the risk of hypertension, hypotension, arrhythmia, and myocarditis. The incidence of hypotension or arrhythmia associated with PD-1/PD-L1 inhibitors was substantially higher than that associated with placebo. When hypertension is observed in patients receiving PD-1/PD-L1 inhibitors, factors other than ICIs should be considered as potential contributors in the first instance.

## Data availability statement

The original contributions presented in the study are included in the article/**Supplementary Material**. Further inquiries can be directed to the corresponding author.

## Author contributions

CZ: Conceptualization, Data curation, Investigation, Methodology, Software, Writing – original draft. FW: Writing – original draft. WM: Writing – original draft. JZ: Investigation, Supervision, Writing – review & editing.

## Supplementary material

Supplementary Figure 1 The assessment of bias risk in the studies included in this meta-analysis.

Supplementary Figure 2 Funnel plots depicting the risk of hypertension in PD-1/PD-L1 + chemotherapy versus chemotherapy. **(A1)** The risk of hypertension of all-grade: subgroup analysis was conducted according to PD-1/PD-L1. **(A2)** The risk of hypertension of grade 3-5: subgroup analyses were conducted according to PD-1/PD-L1. **(A3)** The risk of hypertension of grade 3-5: subgroup analyses were conducted according to types of tumors. Funnel plot depicting the risk of hypertension in PD-1/PD-L1 versus chemotherapy. **(B)** The risk of hypertension of all-grade: subgroup analysis was conducted according to PD-1/PD-L1. Funnel plot depicting the risk of hypertension in PD-1/PD-L1 versus placebo. **(C)** The risk of hypertension of all-grade: subgroup analysis was conducted according to PD-1/PD-L1.

Supplementary Figure 3 Funnel plots depicting the risk of hypotension in PD-1/PD-L1 + chemotherapy versus chemotherapy. **(A1)** The risk of hypotension of all-grade: subgroup analyses were conducted according to PD-1/PD-L1. **(A2)** The risk of hypotension of all-grade: subgroup analyses were conducted according to types of tumors. **(A3)** The risk of hypotension of grade 3-5: subgroup analysis was conducted according to PD-1/PD-L1. Funnel plot depicting the risk of hypotension in PD-1/PD-L1 versus placebo. **(B)** The risk of hypotension of all-grade: subgroup analysis was conducted according to PD-1/PD-L1. Funnel plot depicting the risk of hypotension in PD-1/PD-L1 versus chemotherapy. **(C)** The risk of hypotension of grade 3-5: subgroup analysis was conducted according to PD-1.

Supplementary Figure 4 Funnel plots depicting the risk of arrhythmia in PD-1/PD-L1 + chemotherapy versus chemotherapy. **(A1)** The risk of arrhythmia of all-grade: subgroup analyses were conducted according to PD-1/PD-L1. **(A2)** The risk of arrhythmia of all-grade: subgroup analyses were conducted according to types of tumors. **(A3)** The risk of arrhythmia of grade 3-5: subgroup analyses were conducted according to PD-1/PD-L1. **(A4)** The risk of arrhythmia of grade 3-5: subgroup analyses were conducted according to types of tumors. Funnel plot depicting the risk of arrhythmia in PD-1/PD-L1 versus chemotherapy. **(B)** The risk of arrhythmia of all-grade: subgroup analysis was conducted according to PD-1/PD-L1.

Supplementary Figure 5 Funnel plot depicting the risk of arrhythmia in PD-1/PD-L1 versus placebo. **(A1)** The risk of arrhythmia of all-grade: subgroup analyses were conducted according to PD-1/PD-L1. **(A2)** The risk of arrhythmia of all-grade: subgroup analyses were conducted according to treatment lines.

Supplementary Figure 6 Funnel plot depicting the risk of myocarditis in PD-1/PD-L1 versus chemotherapy. **(A)** The risk of myocarditis of all-grade: subgroup analysis was conducted according to PD-1/PD-L1. Funnel plot depicting the risk of myocarditis in PD-1/PD-L1 versus placebo. **(B)** The risk of myocarditis of all-grade: subgroup analysis was conducted according to PD-1/PD-L1. Funnel plot depicting the risk of myocarditis in PD-1/PD-L1 + chemotherapy versus chemotherapy. **(C)** The risk of myocarditis of all-grade: subgroup analysis was conducted according to PD-1/PD-L1. Funnel plot depicting the risk of cardiovascular toxicities in PD-1/PD-L1 + CTLA-4 versus PD-1/PD-L1. **(D)** The risk of cardiovascular toxicities of all-grade: subgroup analysis was conducted according to PD-1/PD-L1. Funnel plot depicting the risk of cardiovascular toxicities in PD-1/PD-L1 + CTLA-4 versus chemotherapy. **(E)** The risk of cardiovascular toxicities of all-grade: subgroup analysis was conducted according to PD-1/PD-L1.
