# Supplementary figures and images for: Immune-related cardiovascular toxicities of PD-1/PD-L1 inhibitors in solid tumors: an updated systematic review and meta-analysis
Source: Front Immunol. 2024 Jan 22;15:1255825. doi: 10.3389/fimmu.2024.1255825 (PMC10838997; doi:10.3389/fimmu.2024.1255825)

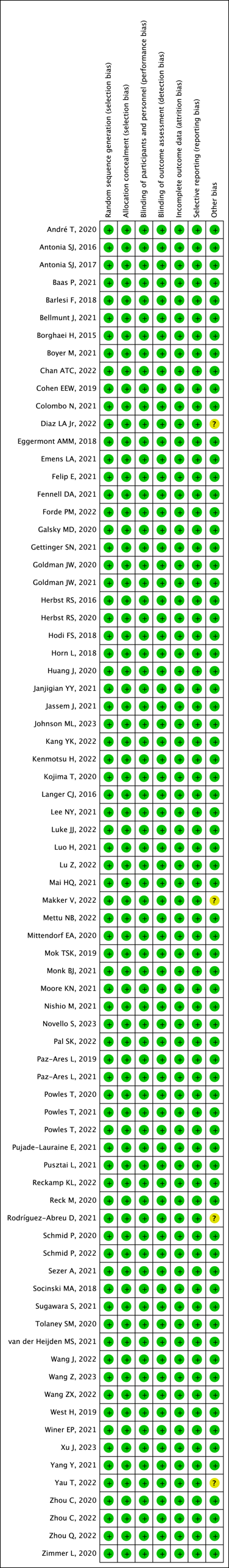

Supplement: Supplementary Figure 1 — The assessment of bias risk in the studies included in this meta-analysis. [file Image_1.jpeg]

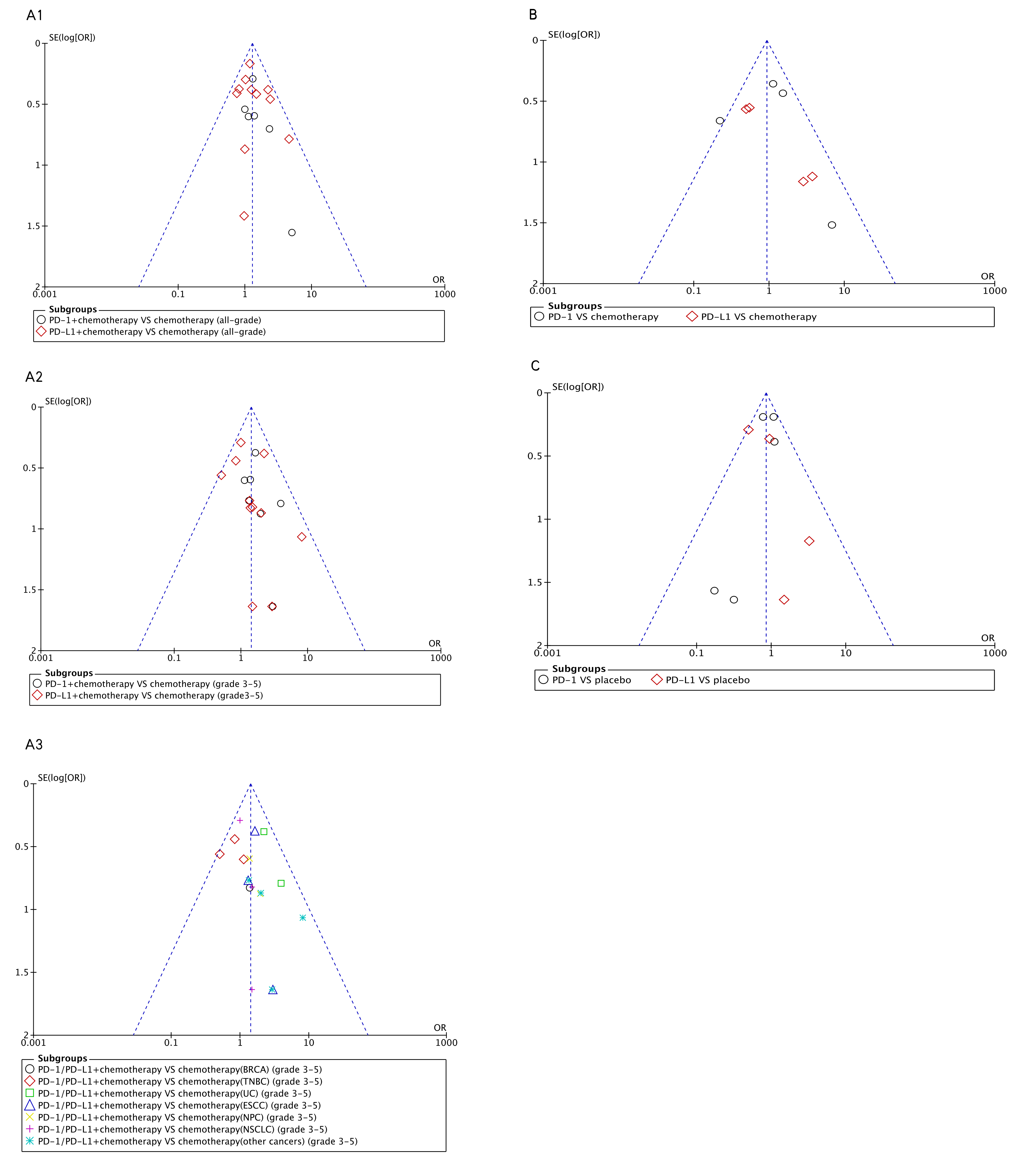

Supplement: Supplementary Figure 2 — Funnel plots depicting the risk of hypertension in PD-1/PD-L1 + chemotherapy versus chemotherapy. (A1) The risk of hypertension of all-grade: subgroup analysis was conducted according to PD-1/PD-L1. (A2) The risk of hypertension of grade 3-5: subgroup analyses were conducted according to PD-1/PD-L1. (A3) The risk of hypertension of grade 3-5: subgroup analyses were conducted according to types of tumors. Funnel plot depicting the risk of hypertension in PD-1/PD-L1 versus chemotherapy. (B) The risk of hypertension of all-grade: subgroup analysis was conducted according to PD-1/PD-L1. Funnel plot depicting the risk of hypertension in PD-1/PD-L1 versus placebo. (C) The risk of hypertension of all-grade: subgroup analysis was conducted according to PD-1/PD-L1. [file Image_2.jpeg]

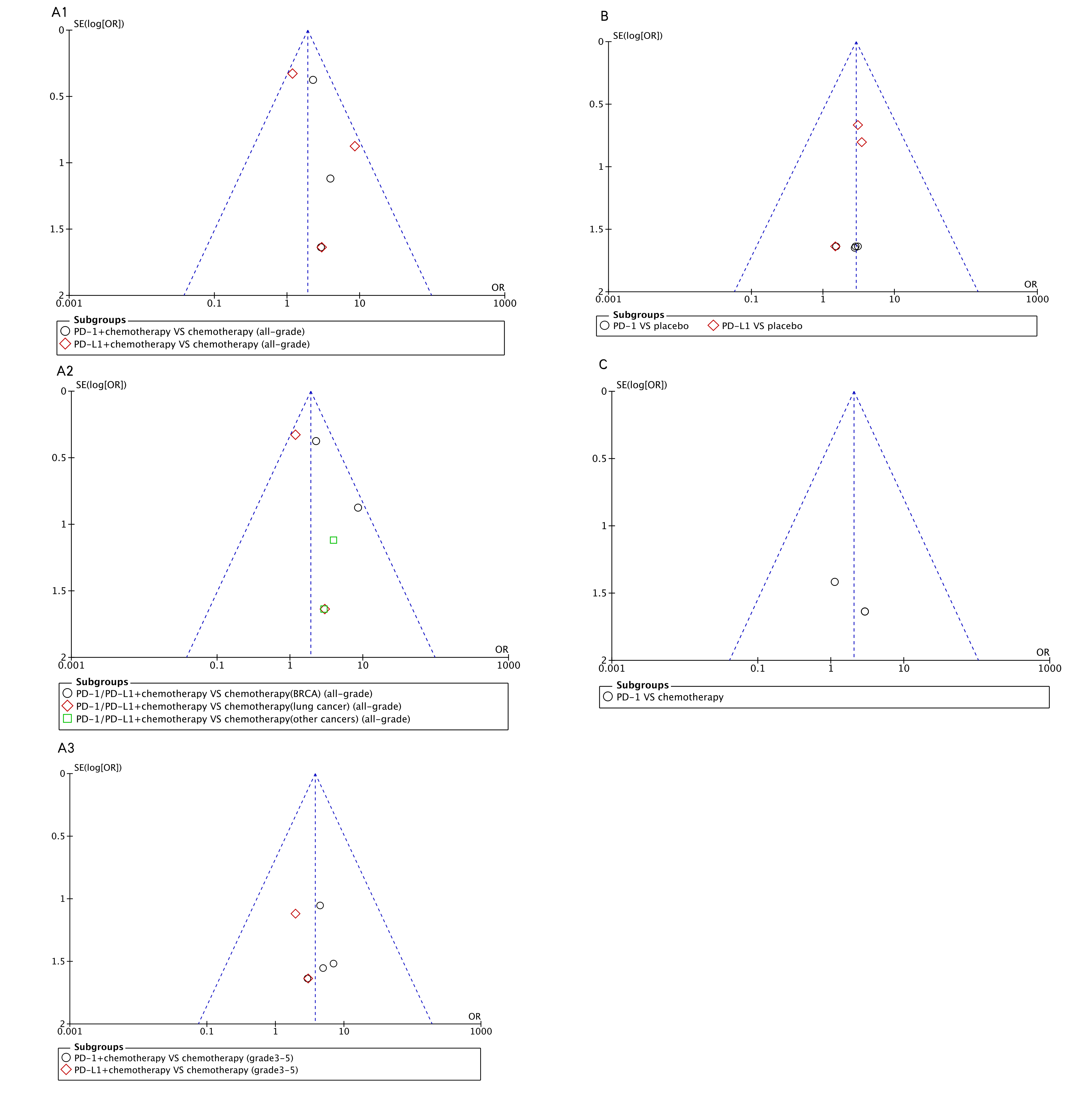

Supplement: Supplementary Figure 3 — Funnel plots depicting the risk of hypotension in PD-1/PD-L1 + chemotherapy versus chemotherapy. (A1) The risk of hypotension of all-grade: subgroup analyses were conducted according to PD-1/PD-L1. (A2) The risk of hypotension of all-grade: subgroup analyses were conducted according to types of tumors. (A3) The risk of hypotension of grade 3-5: subgroup analysis was conducted according to PD-1/PD-L1. Funnel plot depicting the risk of hypotension in PD-1/PD-L1 versus placebo. (B) The risk of hypotension of all-grade: subgroup analysis was conducted according to PD-1/PD-L1. Funnel plot depicting the risk of hypotension in PD-1/PD-L1 versus chemotherapy. (C) The risk of hypotension of grade 3-5: subgroup analysis was conducted according to PD-1. [file Image_3.jpeg]

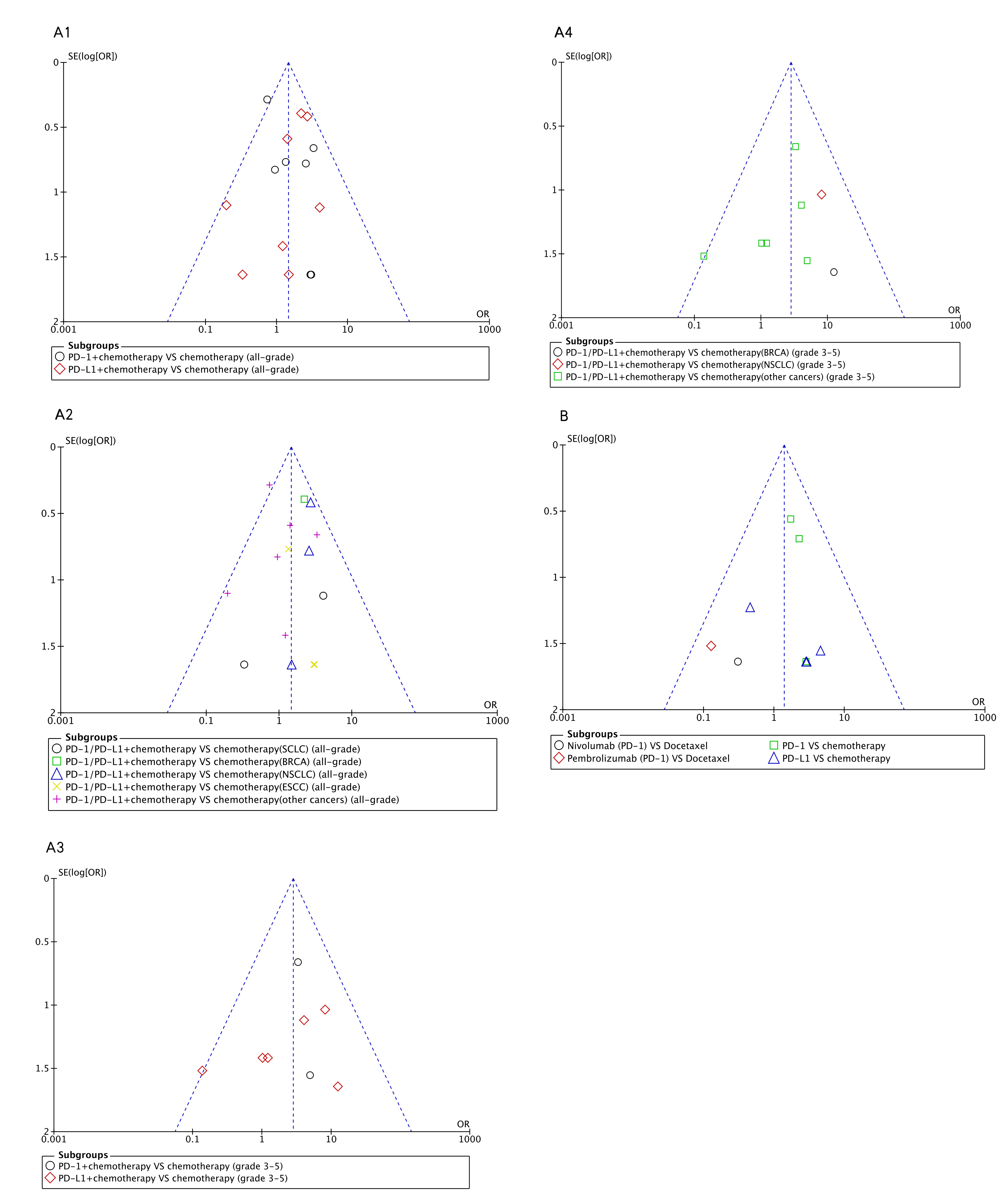

Supplement: Supplementary Figure 4 — Funnel plots depicting the risk of arrhythmia in PD-1/PD-L1 + chemotherapy versus chemotherapy. (A1) The risk of arrhythmia of all-grade: subgroup analyses were conducted according to PD-1/PD-L1. (A2) The risk of arrhythmia of all-grade: subgroup analyses were conducted according to types of tumors. (A3) The risk of arrhythmia of grade 3-5: subgroup analyses were conducted according to PD-1/PD-L1. (A4) The risk of arrhythmia of grade 3-5: subgroup analyses were conducted according to types of tumors. Funnel plot depicting the risk of arrhythmia in PD-1/PD-L1 versus chemotherapy. (B) The risk of arrhythmia of all-grade: subgroup analysis was conducted according to PD-1/PD-L1. [file Image_4.jpeg]

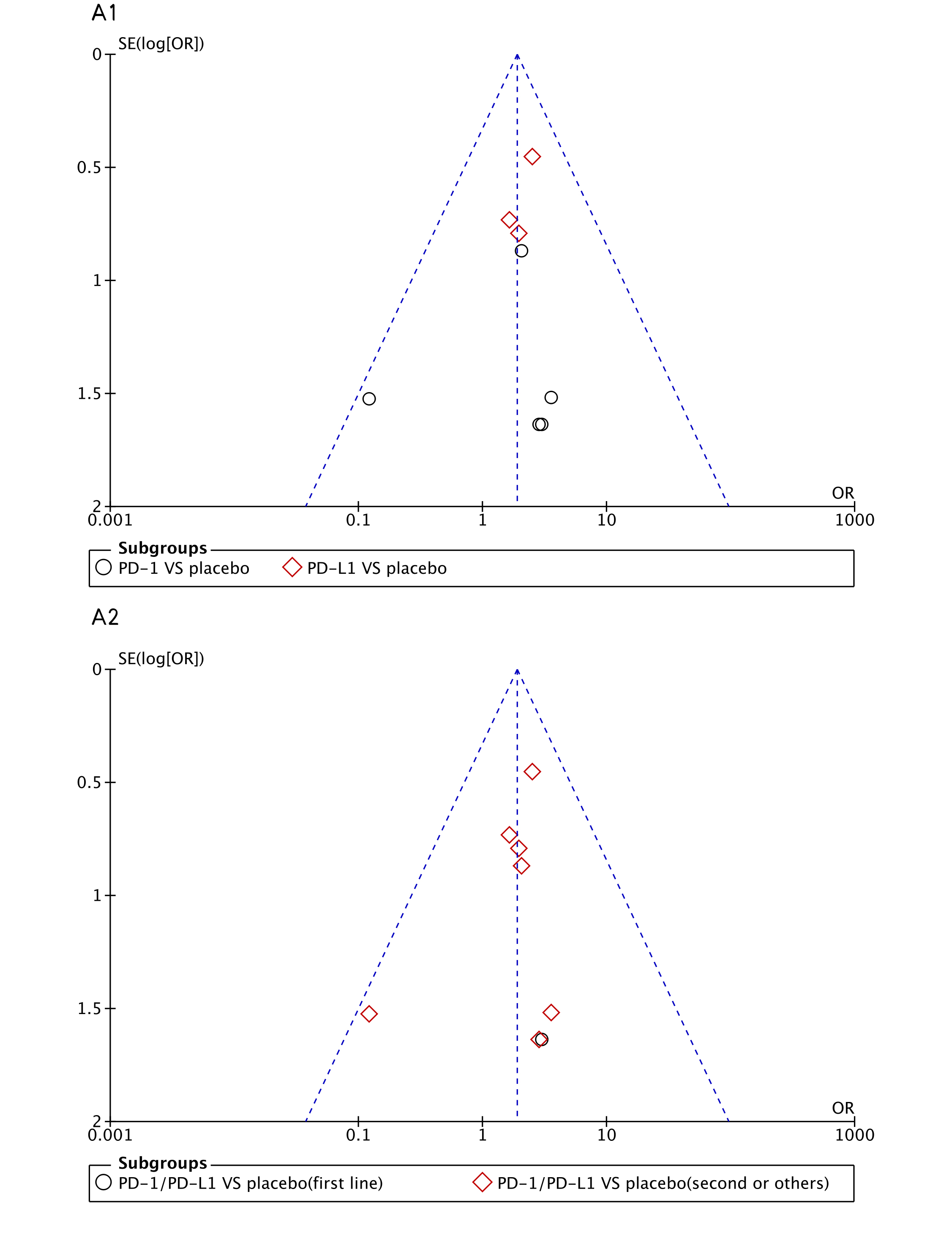

Supplement: Supplementary Figure 5 — Funnel plot depicting the risk of arrhythmia in PD-1/PD-L1 versus placebo. (A1) The risk of arrhythmia of all-grade: subgroup analyses were conducted according to PD-1/PD-L1. (A2) The risk of arrhythmia of all-grade: subgroup analyses were conducted according to treatment lines. [file Image_5.jpeg]

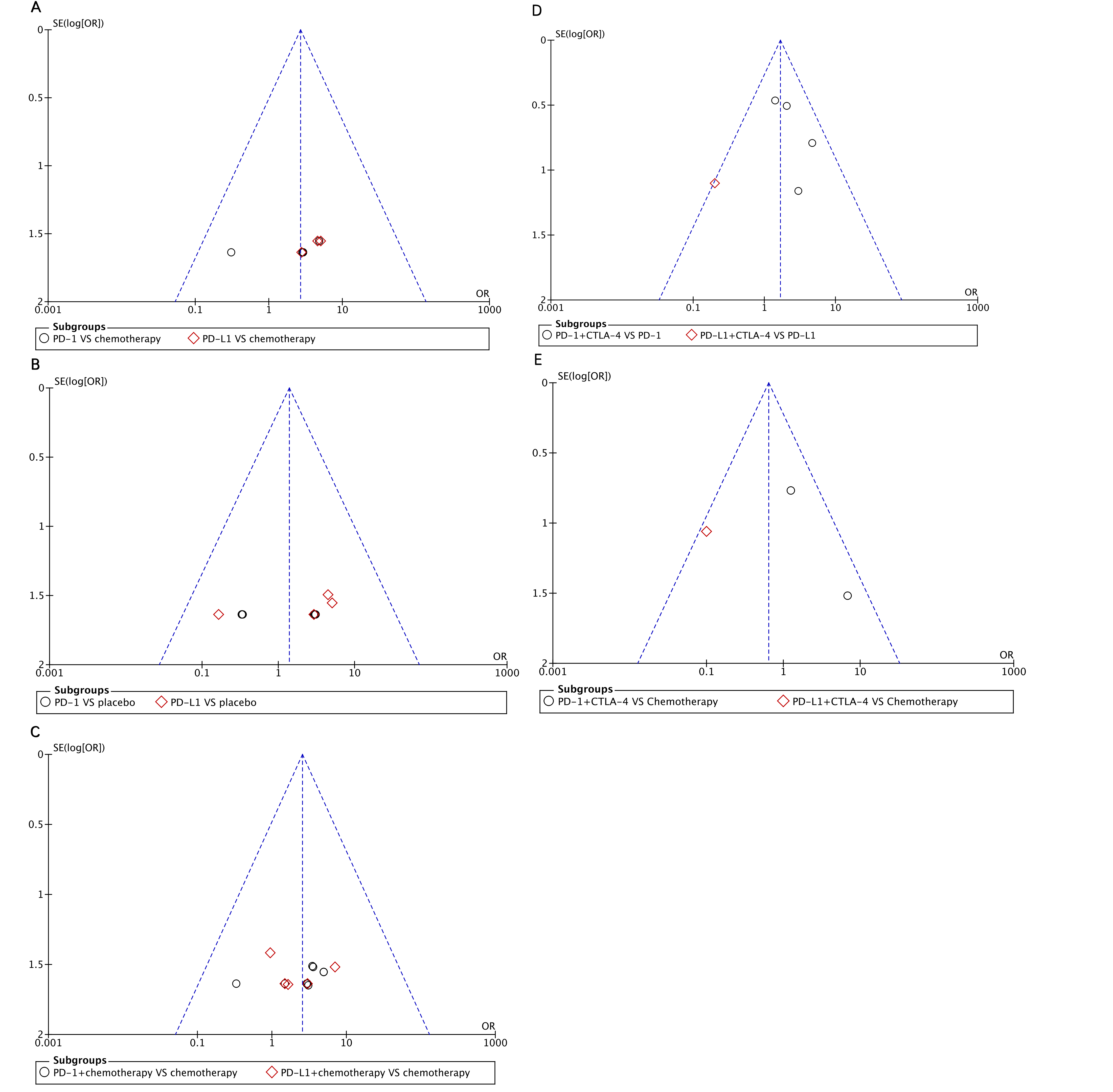

Supplement: Supplementary Figure 6 — Funnel plot depicting the risk of myocarditis in PD-1/PD-L1 versus chemotherapy. (A) The risk of myocarditis of all-grade: subgroup analysis was conducted according to PD-1/PD-L1. Funnel plot depicting the risk of myocarditis in PD-1/PD-L1 versus placebo. (B) The risk of myocarditis of all-grade: subgroup analysis was conducted according to PD-1/PD-L1. Funnel plot depicting the risk of myocarditis in PD-1/PD-L1 + chemotherapy versus chemotherapy. (C) The risk of myocarditis of all-grade: subgroup analysis was conducted according to PD-1/PD-L1. Funnel plot depicting the risk of cardiovascular toxicities in PD-1/PD-L1 + CTLA-4 versus PD-1/PD-L1. (D) The risk of cardiovascular toxicities of all-grade: subgroup analysis was conducted according to PD-1/PD-L1. Funnel plot depicting the risk of cardiovascular toxicities in PD-1/PD-L1 + CTLA-4 versus chemotherapy. (E) The risk of cardiovascular toxicities of all-grade: subgroup analysis was conducted according to PD-1/PD-L1. [file Image_6.jpeg]
